# Major adverse cardiovascular and limb events in patients with diabetes and concomitant peripheral artery disease treated with sodium glucose cotransporter 2 inhibitor versus dipeptidyl peptidase-4 inhibitor

**DOI:** 10.1186/s12933-020-01118-0

**Published:** 2020-09-30

**Authors:** Hsin-Fu Lee, Shao-Wei Chen, Jia-Rou Liu, Pei-Ru Li, Lung-Sheng Wu, Shang-Hung Chang, Yung-Hsin Yeh, Chi-Tai Kuo, Yi-Hsin Chan, Lai-Chu See

**Affiliations:** 1 The Cardiovascular Department, Chang Gung Memorial Hospital, Linkou, Taoyuan 33305 Taiwan; 2 College of Medicine, Chang Gung University, Taoyuan, 33302 Taiwan; 3 Graduate Institute of Clinical Medical Sciences, College of Medicine, Chang Gung University, Taoyuan, Taiwan; 4 Division of Cardiology, Department of Internal Medicine, New Taipei City Municipal Tucheng Hospital (Chang Gung Memorial Hospital, Tucheng branch, Taiwan), Taoyuan, Taiwan; 5 Division of Thoracic and Cardiovascular Surgery, Department of Surgery, Linkou Medical Center, Chang Gung Memorial Hospital, Chang Gung University, Taoyuan City, Taiwan; 6 Department of Public Health, College of Medicine, Chang Gung University, No. 259, Wenhua 1st Rd., Guishan Dist, Taoyuan, 33302 Taiwan; 7 Center for Big Data Analytics and Statistics, Chang Gung Memorial Hospital, Taoyuan, Taiwan; 8 Microscopy Core Laboratory, Chang Gung Memorial Hospital, Linkou, Taoyuan, 33305 Taiwan; 9 Biostatistics Core Laboratory, Molecular Medicine Research Center, Chang Gung University, Taoyuan, 33302 Taiwan; 10 Division of Rheumatology, Allergy and Immunology, Department of Internal Medicine, Chang Gung Memorial Hospital, Linkou, Taoyuan, 33305 Taiwan

## Abstract

**Background:**

Whether sodium glucose co-transporter 2 inhibitors (SGLT2i) are associated with a lower risk of cardiovascular as well as adverse lower limb events in patients with type-2 diabetes mellitus (T2DM) and concomitant peripheral artery disease (PAD) is unclear.

We aimed to evaluate the risk of cardiovascular and limb events, and death associated with the use of SGLT2i compared with dipeptidyl peptidase-4 inhibitors (DPP4i) among a longitudinal and national cohort of patients with T2DM.

**Methods:**

In this nationwide retrospective cohort study based on the Taiwan National Health Insurance Research Database, we identified a total of 11,431 and 93,972 consecutive T2DM patients with PAD taking SGLT2i and DPP4i, respectively, from May 1, 2016, to December 31, 2017. We used 1:1 propensity score matching (PSM) to balance covariates across study groups. Patients were followed from the drug index date until the occurrence of clinical outcomes, death, discontinuation of the index drug, or the end of the study period, whichever occurred first.

**Results:**

Overall, 56% and 44% of the patients were treated with dapagliflozin and empagliflozin, respectively. The use of SGLT2i had comparable risks of ischemic stroke and acute myocardial infarction, and was associated with lower risks of congestive heart failure (CHF) [hazard ratio (HR): 0.66; 95% confidence interval (CI) 0.49–0.89; *p* = 0.0062], lower limb ischemia requiring revascularization (HR: 0.73; 95% CI 0.54–0.98; *p* = 0.0367) or amputation (HR: 0.43; 95% CI 0.30–0.62; *p* < 0.0001), and cardiovascular death (HR: 0.67; 95% CI 0.49–0.90; *p* = 0.0089) when compared with the DDP4i group after PSM. The subgroup analysis revealed consistent results for CHF and major adverse limb outcomes for SGLT2i versus DPP4i among patients aged ≥ 75 years, the presence of chronic kidney disease and established cardiovascular disease was consistent with the main analysis.

**Conclusions:**

SGLT2i were associated with lower risks of CHF and adverse lower limb events compared with DPP4i among patients with T2DM and PAD in real-world practice.

## Background

Sodium-glucose co-transporter-2 inhibitors (SGLT2i) have shown benefits for different endpoints, such as renal outcomes, heart failure, and major cardiovascular (CV) events among patients with type-2 diabetes mellitus (T2DM) treated with antihyperglycemic agents [1–4]. Although the two available trials with dapagliflozin and empagliflozin did not report a significant increase in amputations, the Canagliflozin Cardiovascular Assessment Study (CANVAS) program indicated a higher rate of amputations in the canagliflozin group compared with the placebo group (6.3 vs. 3.4 patients per 1000 patient-years) [1–3]. Nevertheless, the CANVAS results raised concerns regarding the suitability of SGLT2i for patients with T2DM with a high risk of amputation, such as those with concomitant peripheral artery disease (PAD). A few observational studies have investigated the association of SGLT2i with the risk of lower limb amputation; however, these studies have reported inconsistent and conflicting findings. For example, Yuan et al. reported no increased risk of amputations (hazard ratio [HR] 0.98; 95% confidence interval [CI] 0.68–1.41); Adimadhyam et al. reported increased risk (HR 1.38, 95% CI 0.83–2.31); and Udell et al. reported an increased risk (HR 1.99, 95% CI 1.12–3.51) for SGLT2i treatment compared with nonSGLT2i agents [5–7].

T2DM is a major risk factor for CV disease and PAD, and the prevalence of PAD in patients with T2DM has been estimated to reach 20% [8–10]. Patients with T2DM and concomitant PAD have an increased risk of CV events and amputation compared with those without PAD [11]. Subgroup analyses of the landmark studies on empagliflozin revealed consistent CV benefits in patients with T2DM and concomitant PAD without an increased risk of amputation [12]. However, real-world data on the effectiveness, safety, and limb outcomes for such a specific population treated with SGLT2i are scarce. Dipeptidyl peptidase-4 inhibitors (DPP4i) improve glycemic control by increasing the serum levels of glucagon-like peptide 1 (GLP-1) and exhibit a neural effect in CV composite outcomes, that are clinically widely prescribed as second-line agents in the management of hyperglycemia for patients with T2DM [13, 14]. Our study investigated the outcomes of patients with T2DM and concomitant PAD treated with SGLT2i compared with those treated with DPP4i in a large, real-world setting.

## Methods

### Study population

This retrospective nationwide cohort study analyzed data from the Taiwan National Health Insurance (NHI) Research Database (NHIRD), which contains detailed health-care information for more than 23 million enrollees with a > 99% coverage rate of residents of Taiwan [15]. This study was approved by the Institutional Review Board of Chang Gung Medical Foundation, Taiwan (104-8079B and 201801427B0). Informed consent was waived because the original identification number of each patient in the NHIRD had been encrypted and de-identified to protect their privacy.

### Study cohort

**Fig. 1:**
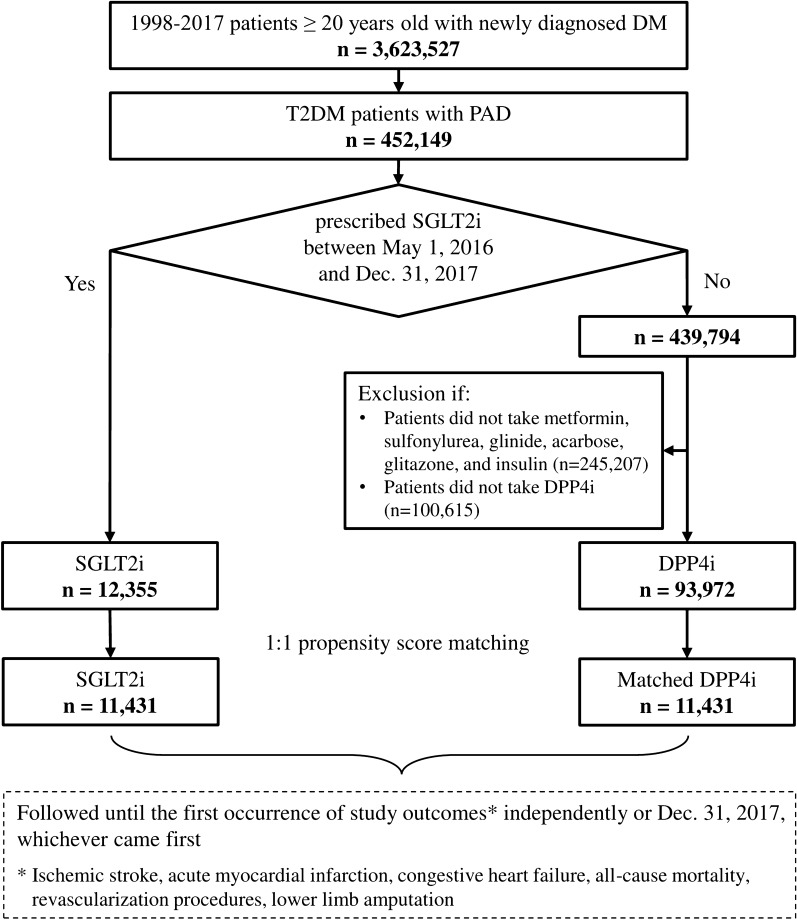
Enrollment of patients with concomitant type-2 diabetes mellitus (T2DM) and peripheral artery disease (PAD). From May 1, 2016 to December 31, 2017, a total of 11,431 patients with T2DM and comorbid PAD treated with sodium-glucose co-transporter-2 inhibitors (SGLT2i) and 11,431 1:1 propensity score matched patients treated with dipeptidyl peptidase-4 inhibitors (DPP4i) were enrolled in the present study. Abbreviations: *DPP4i* dipeptidyl peptidase-4 inhibitor, *PAD* peripheral artery disease; *SGLT2i *sodium-glucose co-transporter-2 inhibitor, *T2DM* type 2 diabetes mellitus

The study identified a total of 3,623,527 patients with T2DM diagnosed using *International Classification of Diseases (ninth revision) Clinical Modification* (ICD-9-CM) codes (250) between January 1, 1998 and December 31, 2015, or *ICD-10-CM codes* (E10.0, E10.1, E10.9, E11.0, E11.1, and E11.9) between January 1, 2016 and December 31, 2017. To identify patients with T2DM who had diagnoses indicating PAD, patients with PAD were required to fulfill with at least one of the following the diagnoses or treatments, which have been registered using medical records, ICD-9-CM or ICD-10-CM diagnostic codes, or ICD-9/10-CM procedural codes (Additional file 1: Table S1). Among the 452,149 patients with T2DM and concomitant PAD, 12,355 patients received first prescriptions of SGLT2i (empagliflozin and dapagliflozin; approval date in Taiwan: May 1, 2016) between May 1, 2016 and December 31, 2017. Canagliflozin has not been included in the present study because it is approved after March 1, 2018 in Taiwan. Of the other 439,794 patients not receiving SGLT2i treatments, 93,972 patients received first prescriptions for DPP4i (saxagliptin, sitagliptin, linagliptin, or alogliptin) during the same period. Patients with T2DM are not allowed to use SGLT2i and DPP4i simultaneously according to Taiwan’s NHI regulations. For each study group, the index date was defined as the first date of prescription for SGLT2i or DPP4i after May 1, 2016. The follow-up period was from the index date until the independent occurrence of any study outcome, discontinuation of the index drug, or end date of the study period (December 31, 2017), whichever occurred first. The flowchart of study enrollment is summarized in Fig. 1.

### Covariates and study outcomes

Baseline covariates were obtained from all claim records with diagnoses, procedures, or medication codes prior to the index date. A history of all prescription medications was confined to medications used at least once within 3 months before the index date. We reported the following outcomes in the present study: (i) ischemic stroke (IS), (ii) acute myocardial infarction (AMI), (iii) congestive heart failure (CHF), (iv) lower limb ischemia requiring revascularization, (v) lower limb amputation, (vi) all-cause mortality, and (vii) cardiovascular mortality. All study outcomes should be the primary discharge diagnosis to avoid misclassification. The diagnostic codes of the NHIRD were shifted from the ICD-9-CM to ICD-10-CM after January 1, 2016. The ICD-9-CM and ICD-10-CM codes used to identify study outcomes along with the baseline covariates are summarized in Additional file 1: Tables S1 and S2.

### Statistical analysis

The propensity score matching (PSM) method, which simulates the design of a randomized clinical trial for observational cohort data by forming matched sets of treated and untreated subjects who share a similar value of the propensity score [16], was used to compare the study outcomes between the SGLT2i and DPP4i group. We calculated propensity score, the predicted probability of treatment conditional on all the covariates in Table 1, by using the generalized boosted model (GBM). The GBM involves an iterative process with multiple regression trees to capture complex and nonlinear relationships between treatment assignment and the pretreatment covariates without over-fitting the data and leading the best balance across study groups [17]. The PSM ratio between the SGLT2i users and DPP4i users was 1:1 without replacement and nearest neighbor matching within a caliper width (8-to-1 digit matching) [18]. The balance of potential confounders at the baseline (index date) between study groups was assessed using the absolute standardized mean difference (ASMD) rather than statistical testing because balance is a property of the sample and not of the underlying population. An ASMD value of ≤ 0.1 would indicate a nonsignificant difference in potential confounders between the two study groups [19]. Incidence rates were estimated using the total number of study outcomes during the follow-up period divided by person-years at risk. The risk of study outcomes occurring over the follow-up duration for SGLT2i versus DPP4i (reference) was obtained using survival analysis (Kaplan–Meier method and log-rank test for univariate analysis and Cox proportional hazards model for multivariate analysis), and they were presented as HRs with 95% CIs. Statistical significance was defined as a *p* value of < 0.05. All statistical analyses were performed using SAS 9.4 (SAS Institute Inc., Cary, NC, USA).

**Table 1:** Clinical characteristics of patients with concomitant type-2 diabetes mellitus (T2DM) and peripheral artery disease (PAD) treated with sodium-glucose co-transporter-2 inhibitors (SGLT2i) and dipeptidyl peptidase-4 inhibitors (DPP4i) before and after propensity score matching (PSM)

	Before PSM			After PSM		
	SGLT2i	DPP4i	ASMD	SGLT2i	DPP4i	ASMD
	(n = 12,355)	(n = 93,972)		(n = 11,431)	(n = 11,431)	
Baseline characteristics						
Age (years)						
Mean	64.3 ± 10.6	70.5 ± 11.3	0.5639	64.7 ± 10.7	65.1 ± 14.5	0.0331
< 65	6188 (50.08%)	27900 (29.69%)	0.5385	5487 (48.00%)	5662 (49.53%)	0.0716
65–74	3990 (32.29%)	29360 (31.24%)		3789 (33.15%)	3677 (32.17%)	
75–84	1861 (15.06%)	26506 (28.21%)		1839 (16.09%)	1791 (15.67%)	
≧ 85	316 (2.56%)	10206 (10.86%)		316 (2.76%)	301 (2.63%)	
Male	6167 (49.92%)	44738 (47.61%)	0.0462	5603 (49.02%)	5660 (49.51%)	0.0100
Chronic lung disease	376 (3.04%)	3852 (4.10%)	0.0569	339 (2.97%)	344 (3.01%)	0.0026
Chronic kidney disease	3276 (26.52%)	37701 (40.12%)	0.2917	3117 (27.27%)	2997 (26.22%)	0.0237
Congestive heart failure	407 (3.29%)	5279 (5.62%)	0.1128	354 (3.10%)	351 (3.07%)	0.0015
Hypertension	10530 (85.23%)	84307 (89.72%)	0.1358	9779 (85.55%)	9730 (85.12%)	0.0121
Dyslipidemia	11062 (89.53%)	80720 (85.90%)	0.1110	10208 (89.30%)	10199 (89.22%)	0.0025
Previous stroke	1159 (9.38%)	14043 (14.94%)	0.1708	1102 (9.64%)	1.036 (9.06%)	0.0198
Ischemic heart disease	2595 (21.00%)	18827 (20.03%)	0.0240	2176 (19.04%)	2172 (19.00%)	0.0009
Gout	3823 (30.94%)	32206 (34.27%)	0.0711	3562 (31.16%)	3532 (30.90%)	0.0057
Malignancy	826 (6.69%)	8644 (9.20%)	0.0930	776 (6.79%)	782 (6.84%)	0.0021
History of bleeding	96 (0.78%)	1639 (1.74%)	0.0868	95 (0.83%)	86 (0.75%)	0.0089
PCI	1570 (12.71%)	11113 (11.83%)	0.0269	1277 (11.17%)	1256 (10.99%)	0.0059
CABG	296 (2.40%)	2547 (2.71%)	0.0199	253 (2.21%)	267 (2.34%)	0.0082
History of diabetic ulcer	161 (1.30%)	2117 (2.25%)	0.0719	148 (1.29%)	183 (1.60%)	0.0256
Baseline medications						
Use of APT	5506 (44.56%)	41766 (44.45%)	0.0024	4969 (43.47%)	4903 (42.89%)	0.0117
Use of NSAIDs	3516 (28.46%)	25954 (27.62%)	0.0187	3299 (28.86%)	3260 (28.52%)	0.0075
Use of PPI	859 (6.95%)	8454 (9.00%)	0.0755	820 (7.17%)	781 (6.83%)	0.0134
Use of ACEI/ARB	7970 (64.51%)	57265 (60.94%)	0.0739	7299 (63.85%)	7305 (63.91%)	0.0011
Use of amiodarone	202 (1.63%)	2564 (2.73%)	0.0749	197 (1.72%)	205 (1.79%)	0.0053
Use of dronedarone	14 (0.11%)	145 (0.15%)	0.0112	9 (0.08%)	13 (0.11%)	0.0113
Use of beta-blocker	4703 (38.07%)	34399 (36.61%)	0.0302	4222 (36.93%)	4166 (36.44%)	0.0102
Use of verapamil/diltiazem	688 (5.57%)	5454 (5.80%)	0.0102	615 (5.38%)	601 (5.26%)	0.0055
Use of digoxin	247 (2.00%)	2125 (2.26%)	0.0182	220 (1.92%)	220 (1.92%)	0.0000
Use of statin	8469 (68.55%)	51472 (54.77%)	0.2862	7609 (66.56%)	7651 (66.93%)	0.0078
Use of metformin	6910 (55.93%)	33242 (35.37%)	0.4217	6403 (56.01%)	6484 (56.72%)	0.0143
Use of sulfonylurea	8282 (67.03%)	50171 (53.39%)	0.2815	7630 (66.75%)	7751 (67.81%)	0.0226
Use of glinide	811 (6.56%)	11886 (12.65%)	0.2076	785 (6.87%)	753 (6.59%)	0.0112
Use of acarbose	2194 (17.76%)	12488 (13.29%)	0.1236	1889 (16.53%)	1870 (16.36%)	0.0045
Use of glitazone	2617 (21.18%)	9538 (10.15%)	0.3071	2244 (19.63%)	2305 (20.16%)	0.0134
Use of insulin	3841 (31.09%)	26103 (27.78%)	0.0727	3433 (30.03%)	3326 (29.10%)	0.0205
Use of loop diuretics	1286 (10.41%)	14791 (15.74%)	0.1586	1191 (10.42%)	1170 (10.24%)	0.0060
Use of MRA	663 (5.37%)	5025 (5.35%)	0.0008	592 (5.18%)	617 (5.40%)	0.0098
Use of ARNI	16 (0.13%)	28 (0.03%)	0.0353	7 (0.06%)	10 (0.09%)	0.0096

*ACEI* angiotensin-converting-enzyme inhibitor, *APT* antiplatelet agent, *ARB* angiotensin II receptor antagonists, *ARNI* angiotensin receptor neprilysin inhibitor, *ASMD* absolute standardized mean difference, *CABG* coronary artery bypass graft, *DDP4i* dipeptidyl peptidase-4 inhibitors, *DM* diabetes mellitus, *MRA* mineralocorticoid receptor antagonist, *NSAIDs* nonsteroid anti-inflammatory drugs, *PAD* peripheral artery disease, *PCI* percutaneous coronary intervention, *PPI* proton pump inhibitor, *PSM* propensity score matching, *SGLT2i* sodium-glucose co-transporter-2 inhibitors, *T2DM* type-2 diabetes mellitus

## Results

### Baseline characteristics of SGLT2i and DDP4i groups

Among the 452,149 patients with T2DM and concomitant PAD, a total of 12,355 and 93,972 were treated with SGLT2i and DDP4i, respectively, from May 1, 2016 to December 31, 2017 (Fig. 1). The mean follow-up periods were 0.96 ± 0.57 and 0.66 ± 0.45 years for SGLT2i and DDP4i, respectively. In the SGLT2i group, 6,915 (56.0%) and 5,440 (44.0%) patients were treated with dapagliflozin and empagliflozin, respectively. In the DDP4i group, 29,782 (31.7%), 24,833 (26.4%), 28,534 (30.4%), 10,636 (11.3%), and 187 (0.2%) patients were treated with sitagliptin, vildagliptin, linagliptin, saxagliptin, and alogliptin, respectively. Before PSM, the SGLT2i group was younger and had a lower prevalence of chronic kidney disease (CKD), hypertension, CHF, hypertension, and stroke history compared with the DDP4i group. The SGLT2i group had a higher rate of dyslipidemia, higher rate of prescriptions for statins, metformin, sulfonylurea, acarbose, and glitazones and a lower rate of prescriptions for glinides. Both study groups were well balanced in all characteristics after PSM (all ASMD < 0.1) (Table 1).

### Main analysis of SGLT2i versus DDP4i

**Fig. 2:**
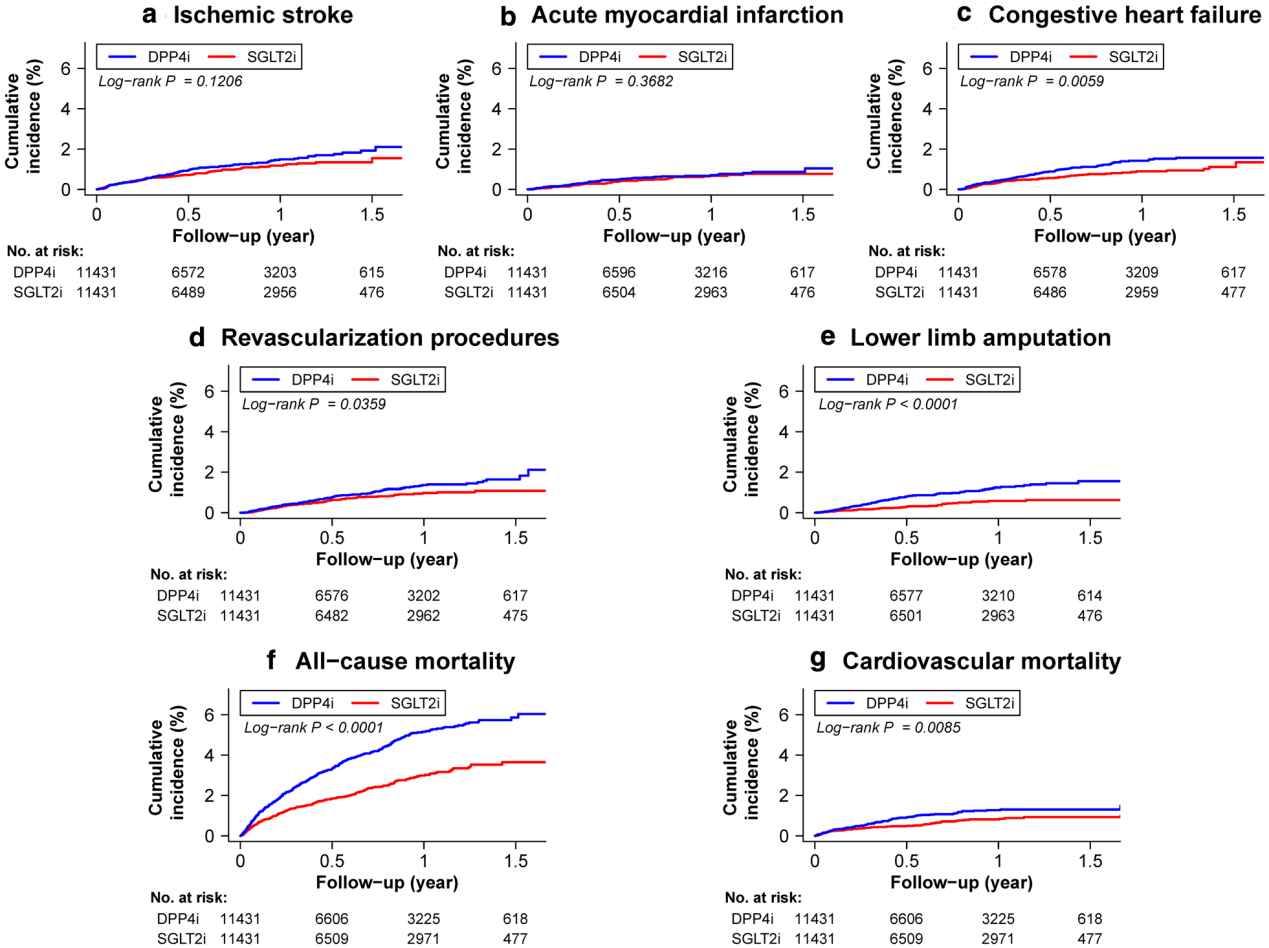
Cumulative incidence curves of outcomes for patients with concomitant type-2 diabetes mellitus (T2DM) and peripheral artery disease (PAD) treated with SGLT2i versus DPP4i after propensity score matching (PSM). Cumulative incidence curves of effectiveness outcomes including ischemic stroke (IS) (**a**), acute myocardial infarction (AMI) (**b**), congestive heart failure (CHF) (**c**), lower extremity revascularization (**d**) or amputation (**e**), all-cause mortality (**f**), and cardiovascular mortality (**g**) for patients with T2DM and concomiant PAD taking SGLT2i versus DPP4i after PSM are presented. SGLT2i were associated with lower cumulative risks of CHF, all-cause and cardiovascular mortality, and lower extremity revascularization or amputation compared with DPP4i among patients with T2DM and concomitant PAD. Abbreviations: *AMI* acute myocardial infarction, *CHF* congestive heart failure, *IS* ischemic stroke, *PSM* propensity score matching. Other abbreviations are the same as those in Fig. 1

The SGLT2i group had comparable cumulative risks of IS and AMI compared with the DPP4i group after PSM. The SGLT2i group was associated with a lower cumulative risk of CHF (log-rank *p* = 0.0059), all-cause (log-rank *p* < 0.0001) and cardiovascular mortality (log-rank *p* = 0.0085) compared with the DPP4i group after PSM. Regarding major adverse limb events, the use of SGLT2i was associated with a lower cumulative risk of lower limb revascularization (log-rank *p* = 0.0359) and amputation (log-rank *p* < 0.0001) compared with the use of DPP4i (Fig. 2).

**Fig. 3:**
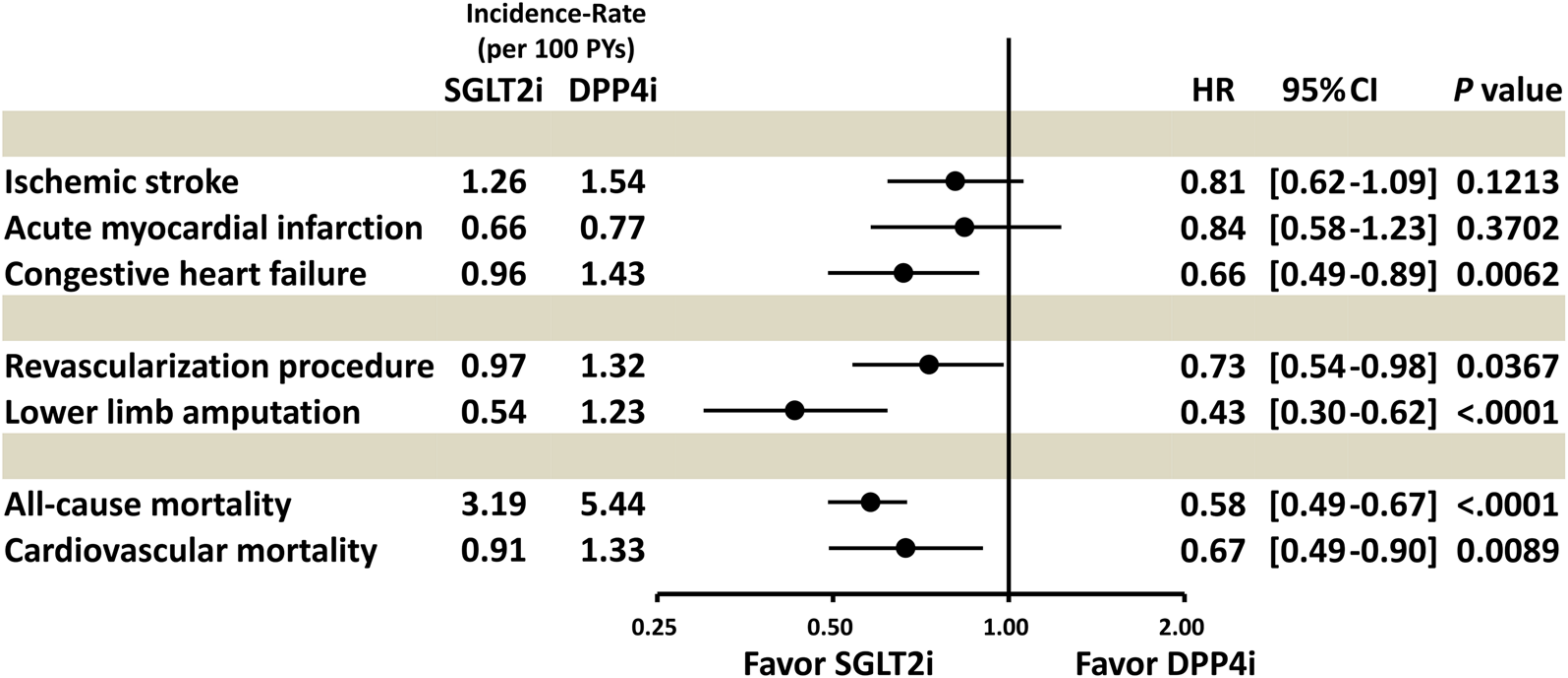
Forest plot of the hazard ratios of clinical outcomes for SGLT2i versus DPP4i among patients with type-2 diabetes mellitus (T2DM) comorbid with peripheral artery disease (PAD) after propensity score matching (PSM). SGLT2i were associated with a comparable risk of thromboembolic events and with lower risks of CHF, lower limb revascularization or amputation, and all-cause or cardiovascular mortality compared with DPP4i among patients with T2DM and concomitant PAD after PSM. Abbreviation: *CI* confidence interval, *HR* hazard ratio. Other abbreviations are the same as those in Figs. 1, 2

The incidence rates (per 100 person-years) of IS (1.26 vs. 1.54, *p* = 0.1213) and AMI (0.66 vs. 0.77, *p* = 0.3702) were comparable between the SGLT2i and DDP4i groups. The SGLT2i group had a significantly lower incidence rate of CHF (0.96 vs. 1.43; HR: 0.66; 95% CI 0.49–0.89; *p* = 0.0062), lower limb ischemia requiring revascularization (0.97 vs. 1.32; HR: 0.73; 95% CI 0.54–0.98; *p* = 0.0367), lower limb amputation (0.54 vs. 1.23; HR: 0.43; 95% CI: 0.30–0.62; *p* < 0.0001), all-cause mortality (3.19 vs. 5.44, HR: 0.58; 95% CI 0.49–0.67; *p* < 0.001), and cardiovascular mortality (0.91 vs. 1.33, HR: 0.67; 95% CI 0.49–0.90; *p* = 0.0089) compared with the DDP4i group (Table 2 and Fig. 3). The use of SGLT2i was not associated with an increased risk of bone fracture or urinary tract infection compared with DDP4i use after PSM (Table 2 and Additional file 1: Table S1**)**.

**Table 2:** Number of events, event rates, and hazard ratio (HR) among patients with type-2 diabetes mellitus and concomitant peripheral artery disease (PAD) using sodium-glucose co-transporter-2 inhibitors (SGLT2i) versus dipeptidyl peptidase-4 inhibitors (DPP4i) after propensity score matching

	SGLT2i		DPP4i		Cox model	
	(n = 11,431)		(n = 11,431)			
Clinical outcome	Number	Incidence rate (per 100 PYs)	Number	Incidence rate (per 100 PYs)	HR (95% CI)	p value
Ischemic stroke (IS)	96	1.26	120	1.54	0.81 (0.62–1.06)	0.1213
Acute myocardial infarction (AMI)	50	0.66	60	0.77	0.84 (0.58–1.23)	0.3702
Congestive heart failure (CHF)	73	0.96	111	1.43	0.66 (0.49–0.89)	0.0062
Lower limb ischemia requiring revascularization	74	0.97	103	1.32	0.73 (0.54–0.98)	0.0367
Lower limb amputation	41	0.54	96	1.23	0.43 (0.30–0.62)	< 0.0001
All-cause mortality	243	3.19	425	5.44	0.58 (0.49–0.67)	< 0.0001
Cardiovascular mortality	69	0.91	104	1.33	0.67 (0.49–0.90)	0.0089
Safety outcome						
Urinary tract infection	331	4.42	297	3.87	1.13 (0.96–1.32)	0.1367
Bone fracture	76	1.00	71	0.91	1.08 (0.78–1.50)	0.6284

*AMI* acute myocardial infarction, *CHF* congestive heart failure, *CI* confidence interval, *DDP4i* dipeptidyl peptidase-4 inhibitors, *HR* hazard ratio, *IS* ischemic stroke, *PAD* peripheral artery disease, *PSM* propensity score matching, *PYs* person-years, *SGLT2i* sodium-glucose co-transporter-2 inhibitors, *T2DM* type-2 diabetes mellitus

### Subgroup analysis of high-risk patients

**Fig. 4:**
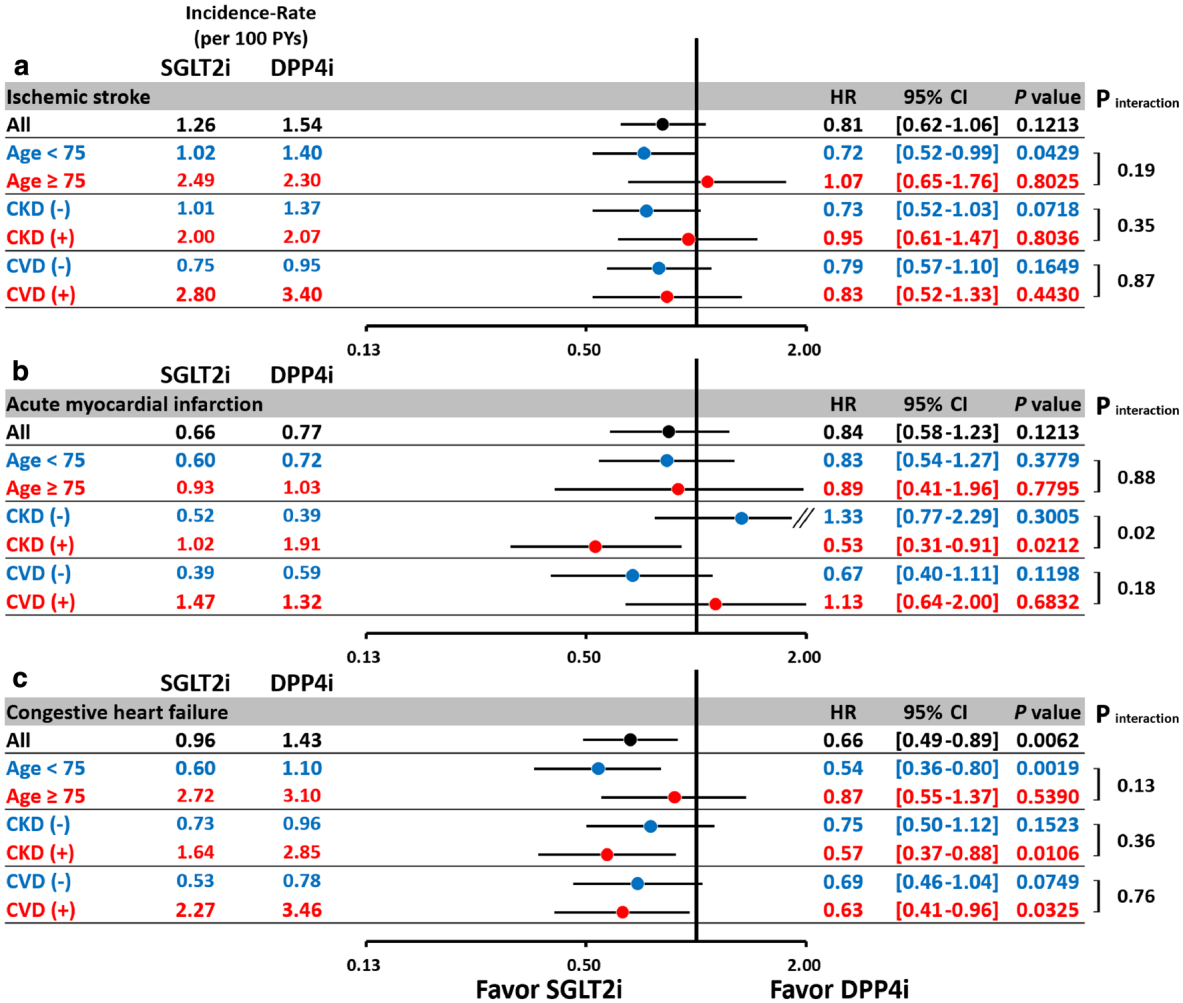
Subgroup analysis of the hazard ratios for the risks of ischemic stroke (IS) (**a**) acute myocardial infarction (AMI) (**b**), and congestive heart failure (CHF) (**c**) for SGLT2i versus DPP4i among T2DM patients with concomitant peripheral artery disease after propensity score matching. In general, the subgroup analysis revealed consistent results for the risks of IS (**a**) AMI (**b**), and CHF (**c**) for SGLT2i versus DPP4i among patients aged $\ge$ 75 years, the presence of chronic kidney disease (CKD) and established CV disease, consistent with the main analysis. The subgroup analysis indicated that SGLT2i reduced the risk of IS and AMI in patients with concomitant CKD but not in patients without CKD (*p* interactions = 0.02). Abbreviations: *CKD* chronic kidney disease, *CV* cardiovascular disease. Other abbreviations as in Figs. 1, 2, 3.

**Fig. 5:**
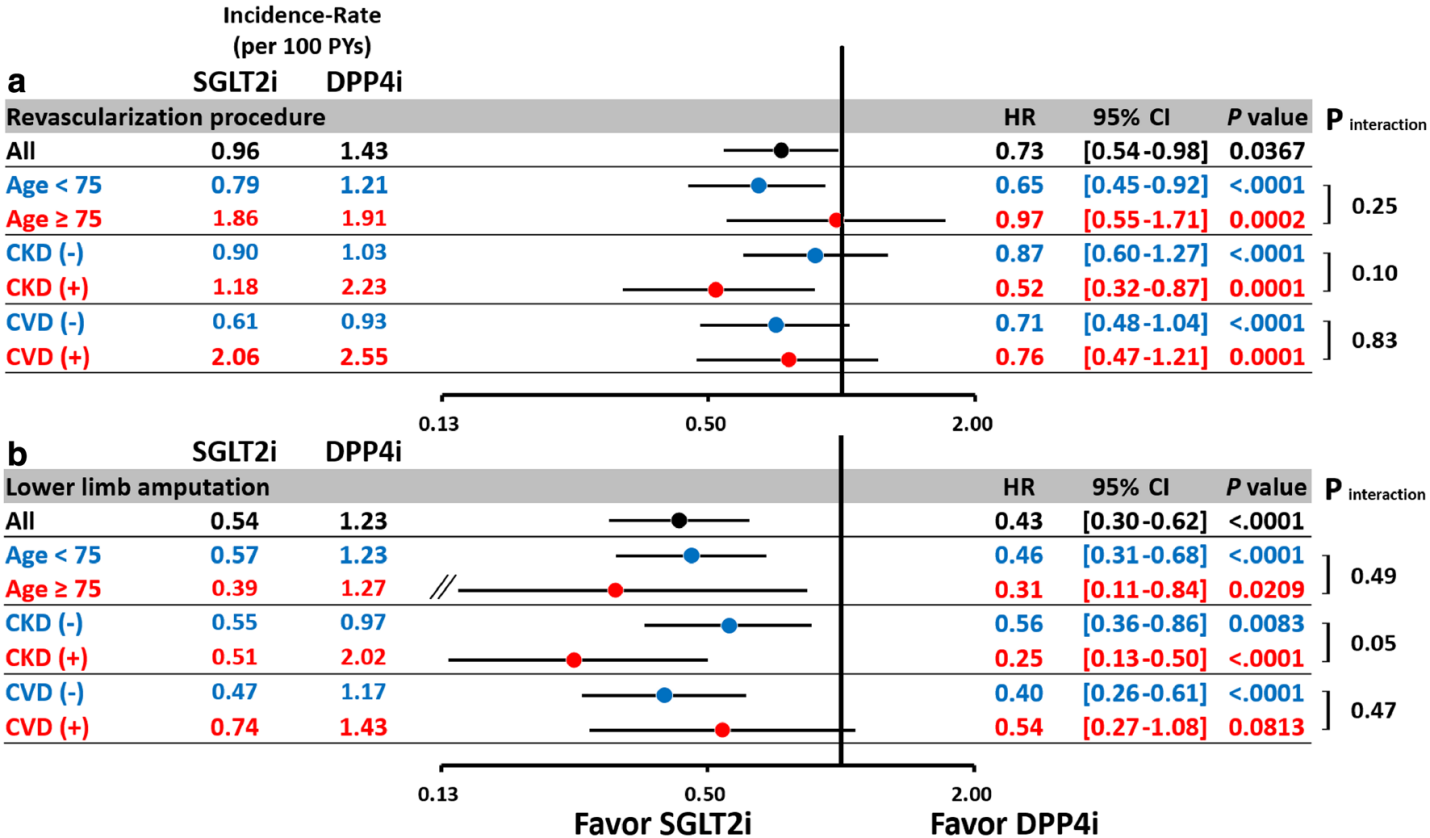
Subgroup analysis of hazard ratios for the risk of major adverse lower limb events including lower limb revascularization procedure **(a)** and amputation (**b**) for SGLT2i versus DPP4i among T2DM patients with concomitant with peripheral artery disease after propensity score matching. The subgroup analysis revealed consistent results for lower limb revascularization (**a**) or amputation (**b**) for SGLT2i versus DPP4i among patients aged ≥ 75 years, the presence of CKD and established CV disease, consistent with the main analysis (all *p* interactions > 0.05). The abbreviations are the same as those in Figs. 1, 2, 3, 4

**Fig. 6:**
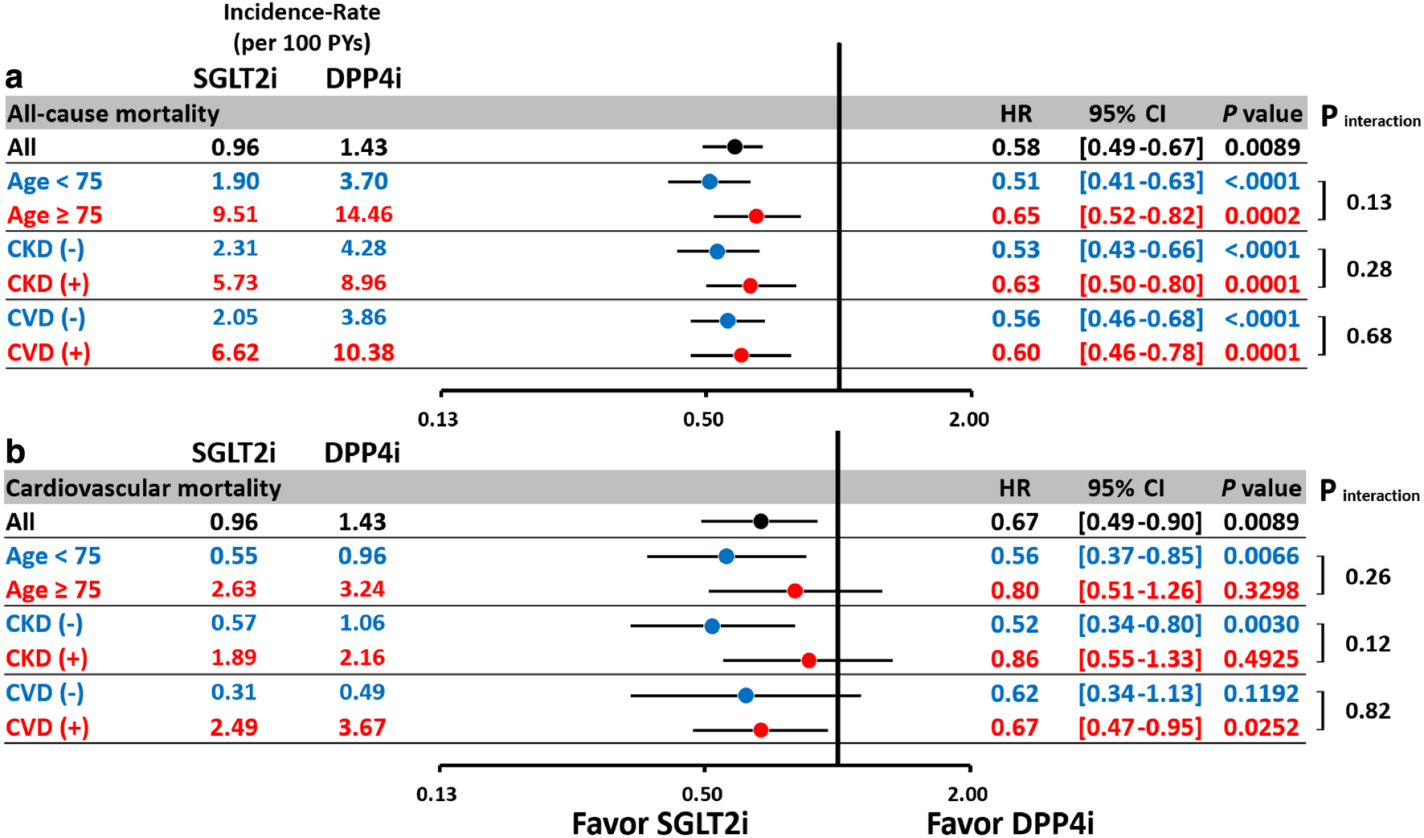
Subgroup analysis of hazard ratios for the risk of all-cause mortality (**a**) and cardiovascular mortality (**b**) for SGLT2i versus DPP4i among T2DM patients with peripheral artery disease after propensity score matching. The subgroup analysis revealed consistent results for all-cause mortality (**a**) and cardiovascular mortality (**b**) for SGLT2i versus DPP4i among patients aged ≥ 75 years, the presence of chronic kidney disease and established CV disease, consistent with the main analysis (all *p* interactions > 0.05). The abbreviations are the same as those in Figs. 1, 2, 3, 4

The subgroup analysis indicated that SGLT2i reduced the risk of AMI in patients with concomitant CKD but not in those without CKD (*p* interaction = 0.02; Fig. 4). In general, the subgroup analysis revealed consistent results for CHF, major adverse limb outcomes, and mortality for SGLT2i versus DPP4i among patients aged ≥ 75 years, the presence of CKD and established CV disease, consistent with the main analysis (Figs. 4, 5,6 ).

## Discussion

To the best of our knowledge, the present study is the first and largest population-based cohort study to investigate the outcomes of patients with T2DM and concomitant PAD treated with SGLT2i compared with those treated with DPP4i. Our findings indicate that SGLT2i was associated with comparable risks of IS and AMI, and significantly lower risks of CHF, lower limb ischemia requiring revascularization or amputation, and all-cause or cardiovascular mortality when compared with DPP4i. This study suggests that SGLT2i is an effective and safe alternative to DPP4i for patients with T2DM and concomitant PAD.

DPP4i improves glycemic control by increasing the serum levels of glucagon-like peptide 1 (GLP-1) through the inhibition of GLP-1 degradation, which indirectly stimulates insulin secretion and enhances beta-cell function. Previous large-scale clinical trials, including EXAMINE, SAVOR-TIMI53, and TECOS, have indicated that the use of DPP4i has a neutral effect in CV composite outcomes for patients with T2DM, except for a higher risk of CHF for those treated with saxagliptin [20–22]. Those clinical studies did not explore the risk of lower limb outcomes for patients with T2DM treated with DPP4i. A previous meta-analysis of the three clinical trials confirmed the benefit of SGLT2i on CHF (HR 0.69; 95% CI 0.61–0.79), all-cause death (HR 0.85; 95% CI 0.78–0.93), and reduced risk of major adverse CV events (composite of myocardial infarction, stroke, and cardiovascular death; HR 0.89, CI 0.83–0.96]) [23]. One large retrospective cohort study also indicated that SGLT2i were associated with lower risks of CHF and death compared with DPP4i in patients with diabetes [24]. Other cohort studies investigating SGLT2i versus other nonSGLT2i antidiabetic agents have consistently reported that SGLT2i reduces the risk of CHF [25–27]. A recent clinical trial also showed the beneficial role of SGLT2i on reducing the risk of HF hospitalization [28, 29].

Patients with T2DM have a higher prevalence of PAD compared with those without T2DM, and patients with T2DM and concomitant PAD have a higher risk of mortality and amputation [11, 30]. However, evidence supporting the benefits of SGLT2i in patients with diabetes and concomitant PAD is limited. The CANVAS program reported a higher rate of amputations in the canagliflozin group compared with the placebo group (0.63 vs. 0.34 per 100 person-years, *p* < 0.001), but not in the pivot studies of empagliflozin (0.65 vs. 0.65 per 100 person-years, *p* = 1.000) and dapagliflozin (1.4 vs. 1.3 per 100 person-years, *p* = 0.53) [1–3]. A clear mechanism explaining why canagliflozin contributes to amputation is lacking; this adverse event may be related to volume depletion, which might accordingly cause circulatory failure in the distal peripheral vasculature [1, 31]. Although a meta-analysis showing patients treated with SGLT2i without a significant association with increased risk of amputation, a large-scale cohort study revealed that SGLT2is were associated with an increased risk of amputation compared with other antihyperglycemic agents for type 2 diabetes [32, 33]. Subgroup analyses from the pivot study of empagliflozin for patients with T2DM and concomitant PAD also revealed benefits of reduced risks of death and CHF without an increased risk of amputation [12]. Consistent with the data, our results indicated that SGLT2i can reduce the risks of CHF and mortality in such a high-risk population. Notably, SGLT2i (dapagliflozin and empagliflozin) were associated with a lower risk of adverse limb events (lower limb ischemia requiring revascularization and lower limb amputation) compared with DDP4i in our study (0.97 vs. 1.32 per 100 person-years, *p* = 0.0367 and 0.54 vs. 1.23 per 100 person-years, *p* < 0.0001). In the assessment of patients with T2DM and concomitant PAD with a relatively high risk of amputation, the absolute risk of amputation in patients treated with SGLT-2i was similar or lower than those seen in the pivot studies, and there is no increase in the probability of amputation [1–3]. In animal or human studies, SGLT2i have been reported to have many benefits for vasculature, such as improved endothelial function, vasodilatation, and attenuated oxidative stress, suggesting that SGLT2i may be able to halt the progression of atherosclerosis and improve vascular outcomes [34–36]. In addition, SGLT2i had been reported to improve cardiometabolic risk factors than DDP4i [37]. PAD is a manifestation of systemic atherosclerosis, and because SGLT2i could reduce the risk of adverse atherosclerotic events, it may also be beneficial in reducing the risk of adverse limb events for patients with PAD [23, 38, 39]. However, studies investigating SGLT2i in patients with T2DM and concomitant PAD are scarce. A subgroup analysis revealed a trend of a lower risk of lower limb amputation (HR: 0.84, 95% CI 0.54–1.32) in the empagliflozin group among patients with T2DM and concomitant PAD [12]. Because patients with T2DM have a high prevalence of PAD [8–10], further randomized or prospective studies should investigate the effect of SGLT2i on lower limb outcomes in such a high-risk population.

## Limitations

To avoid time-lag bias from the prescriptions of study drugs, which may lead to false positive or negative associations depending on the treatments for patients with early or advanced disease, we selected the same second-line hypoglycemic agents of DDP4i as the comparator in our study [7, 40]. To avoid immortal time bias, our study only included new descriptions of study drugs of SGLT2i or DDP4i without baseline use [40, 41]. Nevertheless, the present study had several limitations. First, although PSM with several variables allowed the matching of baseline comorbidities among the study groups, residual confounding by unmeasured variables and prescribing behavior could not be excluded in this retrospective cohort study. Second, the NHIRD does not contain several crucial types of laboratory data such as body weight, glycohemoglobin (HbA1c), and serum creatinine, all of which are associated with the risk of CV events and death among patients with T2DM [42]. In addition, even with adjustment for CKD, the diagnosis of CKD by coding could not reflect the severity of renal disease, which may interfere with SGLT2i or DDP4i selection for each patient. Third, although we utilized some criteria for the selection of the PAD population, our PAD study patients included only part of the PAD population. Thoroughly screening patients with PAD is difficult because PAD populations are typically underrecognized or undertreated in clinical practice, and the incidence of asymptomatic PAD is higher than that of symptomatic PAD [43, 44]. Fourth, miscoding and misclassification of underlying comorbidities and outcomes registered by each physician were another limitation. Therefore, we only considered primary discharge diagnoses to improve the outcome accuracy. However, minor cardiovascular or limb events without admission may have been missed in the present study. Fifth, we did not analyze canagliflozin because of its approval date after March 1, 2018 in Taiwan. Finally, we only investigated Asian patients, and whether our results can be extrapolated to other races remains unclear.

## Conclusions

Our data indicated that SGLT2i, compared with DDP4i, were associated with lower risks of CHF, lower limb ischemia requiring revascularization or amputation, and all-cause death for patients with T2DM and concomitant PAD. Further prospective studies are necessary to evaluate the effects of SGLT2i on lower limb outcomes among such patients in the future.

## Supplementary information

**Additional file 1: Table S1**. International Classification of Diseases (9th and 10th edition) Clinical Modification (ICD 9-CM and ICD 10-CM) codes used to define comorbidities and clinical outcomes in this study.** Table S2.** International Classification of Diseases (9th and 10th edition) Clinical Modification (ICD 9-CM and ICD 10-CM) codes used to define major adverse limb outcomes in this study.** Table S3.** Number of events, event rates, and hazard ratio (HR) among patients with type-2 diabetes mellitus concomitant with peripheral artery disease using sodium-glucose co-transporter-2 inhibitors (SGLT2i) versus dipeptidyl peptidase-4 inhibitors (DPP4i) before propensity score matching.

## Data Availability

The datasets used in this study were only available from the Health and Welfare Data Center, Taiwan. The SAS programs (codes) involved in this study are available from the corresponding author upon reasonable request.

## Abbreviations

ACEI: Angiotensin-converting enzyme inhibitor
AMI: Acute myocardial infarction
APT: Antiplatelet agent
ARB: Angiotensin receptor blocker
ARNI: Angiotensin receptor-neprilysin inhibitor
ASMD: Absolute standardized mean difference
CABG: Coronary artery bypass graft
CHF: Congestive heart failure
CI: Confidence interval
CKD: Chronic kidney disease;
DM: Diabetes mellitus
DPP4i: Dipeptidyl peptidase-4 inhibitor
HR: Hazard ratio
IS: Ischemic stroke
MRA: Mineralocorticoid receptor antagonist
NSAIDs: Nonsteroid anti-inflammatory drugs
PAD: Peripheral artery disease
PCI: Percutaneous coronary intervention
PPI: Proton pump inhibitor
PSM: Propensity score matching
PYs: Person-years
SGLT2i: Sodium glucose cotransporter-2 inhibitor
T2DM: Type-2 diabetes mellitus
