# Supplementary material for: Major adverse cardiovascular and limb events in patients with diabetes and concomitant peripheral artery disease treated with sodium glucose cotransporter 2 inhibitor versus dipeptidyl peptidase-4 inhibitor
Source: Cardiovasc Diabetol. 2020 Sep 30;19:160. doi: 10.1186/s12933-020-01118-0 (PMC7528264; doi:10.1186/s12933-020-01118-0)
Supplement: Supplementary file 1 — Additional file 1: Table S1. International Classification of Diseases (9th and 10th edition) Clinical Modification (ICD 9-CM and ICD 10-CM) codes used to define comorbidities and clinical outcomes in this study. Table S2. International Classification of Diseases (9th and 10th edition) Clinical Modification (ICD 9-CM and ICD 10-CM) codes used to define major adverse limb outcomes in this study. Table S3. Number of events, event rates, and hazard ratio (HR) among patients with type-2 diabetes mellitus concomitant with peripheral artery disease using sodium-glucose co-transporter-2 inhibitors (SGLT2i) versus dipeptidyl peptidase-4 inhibitors (DPP4i) before propensity score matching. [file 12933_2020_1118_MOESM1_ESM.doc]

**Additional file 1: Table S1**

***International Classification of Diseases (9^th^ and 10^th^ edition) Clinical Modification* (ICD 9-CM and ICD 10-CM) codes used to define comorbidities and clinical outcomes in this** study

| **Disease** | **ICD-9 Codes** | **ICD-10 Codes** | **Diagnosis definition** |
| --- | --- | --- | --- |
| Ischemic stroke | 433, 434, 436 | I63, I64 | Discharge |
| Systemic embolism | 444 | I74 | Discharge |
| [Transient ischemic attack](http://en.wikipedia.org/wiki/Transient_ischemic_attack) | 435 | G45 | Discharge |
| Acute Myocardial infarction | 410 | I21-I23 | Discharge |
| Peripheral arterial disease | 440.0, 440.2, 440.3, 440.8, 440.9, 443, 444.0, 444.22, 444.8, 444.9, 447.9, 440.0, 38.08, 38.18, 38.38, 38.48, 38.68, 38.88, 39.50, 39.7, 39.90, 39.25, 39.26, 39.29, 84.10-84.15, 84.16-84.19 | I70.0, I70.2, I70.9, I70.3, I70.8, I75.89, I70.9, I73.0, I73.1, I73.8, I73.9, I79.1, I79.8, I74.01, I74.09, I74.3, I74.4, I74.5, I74.8, I74.9, I77.9, I70.0, 41, 045, 047, 049, 04B, 04C, 04H, 04J, 04L, 04N, 04P, 04Q, 04R, 04S, 04U, 04V, 04W  Location:(C,D,E,F,H,J,K,L,M,N,P,Q,R,S,T,U,V,W,Y)  0Y67, 0Y68, 0Y6C, 0Y6D, 0Y6F, 0Y6G, 0Y6H, 0Y6J, 0Y6M, 0Y6N, 0Y6P, 0Y6Q, 0Y6R, 0Y6S, 0Y6T, 0Y6U, 0Y6V, 0Y6W, 0Y6X, 0Y6Y | Discharge or Outpatient department ≥2 |
| Ischemic heart disease | 410, 411, 412, 413, 414 | I21-I25 | Outpatient department ≥2 |
| Congestive heart failure | 428 | I11.0, I13.0, I13.2, I42.0, I50, I50.1, I50.9 | Discharge |
| Hypertension | 401, 402 | I10-I16 | Outpatient department ≥2 |
| Diabetes mellitus | 250 | E11-E14 | Outpatient department ≥2 |
| Hyperlipidemia | 272 | E78 | Outpatient department ≥2 |
| Chronic gout | 274.0, 274.10, 274.11, 274.19, 274.81, 274.82, 274.89, 274.9 | M10, M1A | Outpatient department ≥2 |
| Chronic lung disease | 490, 491.0, 491.1, 491.20-491.22, 491.8, 491.9, 492.0, 492.8, 493.00-493.02 493.10-493.12, 493.20-493.22, 493.81, 493.82, 493.90-493.92, 494.0, 494.1, 495.8, 495.9, 496, 500, 502, 503, 504, 505, A323, A325 | J41-J44 | Discharge |
| Chronic kidney disease | 580-589 | I12, I13, N00, N01, N02, N03, N04, N05, N07, N11, N14, N17, N18, N19, Q61 | Outpatient department ≥2 |
| Chronic liver disease | 570, 571, 572 | B150, B160, B162, B190, K704, K72, K766, I85 | Outpatient department ≥2 |
| Malignancy | 140.0-208.9 | C | Outpatient department ≥2 |
| Diabetic ulcer |  | E11.621 | Discharge or Outpatient department ≥2 |
| Diabetic ulcer:  Primary diagnosis of DM plus ulcer of lower limb | 707.1 (Ulcer of lower limb) | L97.901, L97.902, L97.903, L97.904, L97.909, L97.911, L97.912, L97.913, L97.914, L97.919, L97.921, L97.922, L97.923, L97.924, L97.929, L97.101, L97.102, L97.103, L97.104, L97.109, L97.111, L97.112, L97.113, L97.114, L97.119, L97.121, L97.122, L97.123, L97.124, L97.129, L97.201, L97.202, L97.203, L97.204, L97.209, L97.211, L97.212, L97.213, L97.214, L97.219, L97.221, L97.222, L97.223, L97.224, L97.229, L97.301, L97.302, L97.303, L97.304, L97.309, L97.311, L97.312, L97.313, L97.314, L97.319, L97.321, L97.322, L97.323, L97.324, L97.329, L97.401, L97.402, L97.403, L97.404, L97.409, L97.411, L97.412, L97.413, L97.414, L97.419, L97.421, L97.422, L97.423, L97.424, L97.429, L97.501, L97.502, L97.503, L97.504, L97.509, L97.511, L97.512, L97.513, L97.514, L97.519, L97.521, L97.522, L97.523, L97.524, L97.529, L97.801, L97.802, L97.803, L97.804, L97.809, L97.811, L97.812, L97.813, L97.814, L97.819 | Discharge or Outpatient department ≥2 |

**Table S2**

***International Classification of Diseases (9^th^ and 10^th^ edition) Clinical Modification* (ICD 9-CM and ICD 10-CM) codes used to define major adverse limb outcomes in this** study

| Disease | ICD-9 Codes | ICD-10 Codes | Diagnosis definition |
| --- | --- | --- | --- |
| Revascularization  (Procedural codes) | 38.08 (incision of vessel, lower limb arteries) | 041,045,047,049,04B,04C,04H,  04J,04L,04N,04P,04Q,04R,04S,  04U,04V,04W  Location:(C,D,E,F,H,J,K,L,M,N,P,Q,R,S,T,U,V,W,Y) | Discharge |
|  | 38.18 (endarterectomy, lower limb arteries) |  |  |
|  | 38.38 (resection of vessel with anastomosis, lower limb arteries) |  |  |
|  | 38.48 (resection of vessel with replacement, lower limb arteries) |  |  |
|  | 38.68 (other excision of vessel, lower limb arteries) |  |  |
|  | 38.88 (other surgical occlusion of vessel, lower limb arteries) |  |  |
|  | 39.50 (angioplasty or atherectomy of non-coronary vessel) |  |  |
|  | 39.7 (Endovascular repair of vessel) |  |  |
|  | 39.90 (insertion of non-coronary artery stent) |  |  |
|  | 39.25 (aorta-iliac-femoral bypass) |  |  |
|  | 39.26 (other intra-abdominal vascular shunt or bypass) |  |  |
|  | 39.29 (other(peripheral) vascular shunt or bypass) |  |  |
| Amputation | - 1. (amputation of lower limb) | 0Y67, 0Y68, 0Y6C, 0Y6D, 0Y6F, 0Y6G, 0Y6H, 0Y6J, 0Y6M, 0Y6N, 0Y6P, 0Y6Q, 0Y6R, 0Y6S, 0Y6T, 0Y6U, 0Y6V, 0Y6W, 0Y6X, 0Y6Y |  |
|  | 84.10 ~ 84.15 low level amputation (amputation below knee) |  |  |
|  | 84.16 ~ 84.19 high level amputation (knee disarticulation or above) |  |  |

**Table S3**

**Number of events, event rates, and hazard ratio (HR) among patients with type-2 diabetes mellitus concomitant with peripheral artery disease using sodium-glucose co-transporter-2 inhibitors (SGLT2i) versus dipeptidyl peptidase-4 inhibitors (DPP4i) before propensity score matching**

|  | **SGLT-2 inhibitors** | | **DPP-4 inhibitors** | | **Cox model** | |
| --- | --- | --- | --- | --- | --- | --- |
|  | **(n = 12,355)** | | **(n = 93,972)** | |  |  |
| **Clinical outcome** | **Number** | **Incidence rate** | **Number** | **Incidence rate** | **HR (95% CI)** | **P value** |
| **Ischemic stroke** | 104 | 1.28%/year | 1,570 | 1.74%/year | 0.66 (0.54-0.81) | <0.0001 |
| **Acute myocardial infarction** | 56 | 0.69%/year | 1,113 | 1.23%/year | 0.52 (0.40-0.68) | <0.0001 |
| **Congestive heart failure** | 85 | 1.04%/year | 2,076 | 2.30%/year | 0.40 (0.32-0.50) | <0.0001 |
| **Limb ischemia requiring revascularization** | 80 | 0.98%/year | 1,418 | 1.57%/year | 0.58 (0.46-0.72) | <0.0001 |
| **Lower limb amputation** | 44 | 0.54%/year | 1,060 | 1.17%/year | 0.41 (0.30-0.56) | <0.0001 |
| **All-cause mortality** | 250 | 3.06%/year | 9,050 | 9.99%/year | 0.27 (0.24-0.30) | <0.0001 |
| **Cardiovascular mortality** | 72 | 0.88%/year | 2140 | 2.36%/year | 0.32 (0.26-0.41) | <0.0001 |
|  |  |  |  |  |  |  |
| **Safety outcome** |  |  |  |  |  |  |
| **Urinary tract infection** | 347 | 4.32%/year | 3,833 | 4.33%/year | 0.88 (0.79-0.98) | 0.0236 |
| **Bone fracture** | 79 | 0.97%/year | 1,316 | 1.46%/year | 0.64 (0.51-0.81) | 0.0001 |

AMI = acute myocardial infarction; CHF = congestive heart failure; CI = confidence interval; DDP4i = dipeptidyl peptidase-4 inhibitors; HR = hazard ratio; PAD = peripheral artery disease; PSM = propensity score matching; SGLT2i = sodium-glucose cotransporter-2 inhibitors; T2DM = type-2 diabetes mellitus.
